# Maternal BMI and smoking partly explain the association between maternal socio-economic position and offspring asthma

**DOI:** 10.1136/thorax-2025-223330

**Published:** 2025-10-28

**Authors:** Cecilia Lundholm, Emma Caffrey Osvald, Samuel Rhedin, Catarina Almqvist

**Affiliations:** 1 Department of Medical Epidemiology and Biostatistics, Karolinska Institutet, Stockholm, Sweden; 2 Astrid Lindgren Children’s Hospital, Paediatric Allergy and Pulmonology Unit, Karolinska University Hospital, Stockholm, Sweden; 3 Sachsska Children’s and Youth Hospital, Stockholm, Sweden

**Keywords:** asthma, asthma epidemiology, child, paediatric asthma, smoking

## Abstract

**Background:**

Numerous studies have found associations between parental socio-economic position (SEP) and offspring asthma. Obesity and tobacco smoking during pregnancy (SDP) are more prevalent in lower SEP groups and have been identified as risk factors for childhood asthma.

**Aim:**

The aim of this study was to explore and quantify the role of the modifiable risk factors—obesity, body mass index (BMI) and SDP—in the association between maternal SEP and offspring asthma.

**Methods:**

This Swedish register-based cohort study included n=1 265 933 children born between 2006 and 2018, with information on asthma during the third and sixth years of life obtained from the National Patient Register and the Swedish Prescribed Drug Register. Maternal education was used as a measure of SEP. We used logistic regression to estimate exposure-outcome associations and applied the counterfactual approach to mediation analysis to estimate the proportion of the maternal SEP-offspring asthma association that could be explained by maternal obesity, BMI and SDP, with and without adjustment for birth year.

**Result:**

The OR was 1.15 (95% CI 1.13 to 1.16) for the association between maternal education and asthma during the third year of life and slightly lower during the sixth year (OR 1.09, 95% CI 1.07 to 1.10). Out of the excess risks, 20%–30% could be explained by obesity or BMI through mediation and mediated interaction, while 15%–20% could be explained by SDP.

**Conclusion:**

Our results indicate that supporting young women and mothers-to-be to healthy weight and to abstain from tobacco smoking, in particular during pregnancy, could reduce child morbidity and improve health equity in childhood.

**WHAT IS ALREADY KNOWN ON THIS TOPIC**

- There is evidence that children of parents with lower socio-economic position have a higher risk of developing asthma than other children.

**WHAT THIS STUDY ADDS**

- We show that 15%–30% of this excess risk can be explained by the modifiable risk factors of maternal obesity and smoking during pregnancy.

**HOW THIS STUDY MIGHT AFFECT RESEARCH, PRACTICE OR POLICY**

- Our results indicate that supporting mothers-to-be to healthy weight and smoking cessation has the potential to reduce childhood health inequity regarding asthma.

## Introduction

 Social determinants, such as the conditions in which people are born and live, and people’s access to power, money and resources influence health outcomes.^1^ Moreover, children of parents with low socio-economic position (SEP) have worse health outcomes than those whose parents have higher education and income, also in high-income countries with universal healthcare such as Sweden.^2^ Health inequity is also seen in asthma. Numerous studies have observed an association between parental SEP and childhood asthma.^3^

SEP has been measured in many different ways in health research, including housing conditions, education, income, occupation and composite measures.^4 5^ There is no SEP indicator that is the preferred measure for all study aims and all settings.^5^ While education can be viewed as a measure of the knowledge-related assets of a person, income reflects the material assets. Income is more volatile than other measures and can change due to health problems and having children. Education is often completed in early adulthood and influenced by parental characteristics, and thereby it can also be viewed as a generic measure of SEP.^5^ We have previously shown evidence of low education being associated with a higher risk of asthma after 1 year of age in Sweden, but not low income.^6 7^

Social inequalities tend to be persistent also in modern welfare states.^8^ It is therefore important to find downstream factors that can be targeted to reduce common chronic disease such as asthma and other health disparities among children.

The impact of social determinants on a child’s health outcomes is present already in pregnancy, through maternal health, environment and lifestyle. In Sweden, as in many other countries, women are monitored by healthcare professionals during the pregnancy. Pregnant women are also usually concerned about the health of the expected child. Therefore, the pregnancy period could be a window of opportunity to intervene on risk factors affecting both the mother and the expected child.

Maternal smoking^9 10^ and obesity^11 12^ in pregnancy have both been identified as risk factors for offspring asthma, both in original articles and systematic reviews. Those are also more commonly seen in women with low education compared with women with higher education.^13^ Mediation analysis can help in finding what factors to target with interventions, by providing estimates of how large a part of the association between SEP and childhood asthma can be explained by those factors and to what extent they interact with SEP.

Our aim was to investigate the role of the modifiable risk factors such as maternal obesity and smoking during pregnancy (SDP) as mediators in the association between maternal education and offspring asthma.

## Methods

In this population and register-based cohort study, we included all children born in Sweden from 1 January 2006 to 31 December 2018 (n=1 367 272) according to the Register of the Total Population (RTP), which holds information on birth date, death date, sex and parents’ identity for all Swedish residents.^14^ Since the mediators of interest were retrieved from the Medical Birth Register (MBR),^15^ we excluded children not recorded in the register (n=55 953). We also excluded children with missing identity of the mother (n=91), mothers not registered as resident in Sweden (n=121), those with missing information on mother’s education (n=30 263), children who emigrated (n=11 453) or died (n=3 458) before age 3 years according to the RTP, leaving us with a cohort of n=1 265 933 children. The registers could be linked unambiguously based on the unique personal identity number given to each person residing in Sweden.^16^

All data we retrieved from the registries were pseudonymised by the register holders.

### Exposure

We used maternal education as a measure of SEP rather than other measures such as income, since we wanted a generic and stable measure of SEP and since we have previously shown that low education, but not low income, was associated with a higher risk of asthma after 1 year of age.^6 7^ This was defined as the mother’s highest attained education registered in the Swedish Longitudinal Integrated Database for Health Insurance and Labour Market Studies (LISA) in the year before the child’s birth. LISA is a national database including data on education, social insurance utilisation, income and employment.^17^ Education was categorised as low or high education, with low education being upper secondary school or less, and high education being tertiary education. If maternal education information was missing for the year before birth, we used the mother’s education from the year the child was born (n=130 667).

### Mediators

As mediators, we used maternal body mass index (BMI) and smoking in early pregnancy, both retrieved from the MBR. The MBR includes information on pregnancies and deliveries for almost all children born in Sweden since 1973, including data on maternal smoking, height (in metres), weight (in kilograms), age and birth outcomes.^15^ BMI was calculated based on height and weight from the first antenatal care visit (BMI=weight/height^2^), used both as a continuous variable and categorised as obesity yes (BMI ≥30 kg/m^2^) or no (BMI <30 kg/m^2^). SDP was reported by the mother at the first antenatal care visit and categorised as yes (≥1 cigarette/day) or no (<1 cigarette/day). The height, weight and smoking variables in the MBR are all considered to have very good validity.^18 19^

### Outcome

Current asthma during the third and sixth years of life was used as outcomes, to capture both viral-induced wheeze that may be intermittent and a more established asthma phenotype. Asthma was defined in accordance with a validated algorithm,^20^ based on outpatient care specialist diagnoses in the National Patient Register (NPR)^21^ and dispensed prescriptions on asthma medication in the Swedish Prescribed Drug Register (SPDR).^22^ The NPR includes information on all inpatient hospital visits since 1987 and approximately 80% of all outpatient specialist care visits since 2001, with data on diagnoses by the International Classification of Diseases (ICD), medical procedures and date of visit.^21^ The SPDR started in 2005 to cover all drug prescriptions that are dispensed at pharmacies in Sweden, including prescription and dispense date, Anatomic Therapeutic Chemical code and amount dispensed.^22^

According to the asthma algorithm,^20^ asthma was defined based on the following criteria:

1. ≥2 dispenses of asthma controller medication, including inhaled corticosteroids (ICS; R03BA), leukotriene receptor antagonist (R03DC03) and/or fixed combinations of ICS and β2-agonists (R03AK06 or R03AK07), regardless of timing for children aged >4.5 years and with ≥14 days between the two dispenses for children aged ≤4.5 years.
2. ≥1 dispensed controller medication and ≥2 of short-acting inhaled β2-agonists (R03AC02, R03AC03, R03AC12, R03AC13) before the third and sixth birthday.
3. ≥3 dispenses of short-acting inhaled β2-agonists within any period of 12 months before the third and sixth birthday.
4. ≥1 specialist care visit with an asthma diagnosis (ICD V.10: J45-J46) in the NPR

The validation study showed positive predictive values for each of the four criteria above for children above 4.5 years of age, but less so for children below 4.5 years. For younger children, we therefore required meeting at least one of criteria (1)–(3) in combination with criteria (4), while for children above 4.5 years, fulfilling any one of criteria (1)–(4) was sufficient.

Current asthma during the third year of life was defined as fulfilling the algorithm before 3 years of age, with at least one specialist care diagnosis of asthma or at least one filled prescription on or after the second birthday and before the third birthday. Similarly, current asthma during the sixth year of life was defined as fulfilling the algorithm before the sixth birthday and having at least one filled prescription/asthma diagnosis between the fifth and sixth birthdays. Children who emigrated (n=11 896) or died (n=306) before the sixth birthday were excluded from analyses regarding asthma during the sixth year of life.

### Other variables

**Figure 1:**
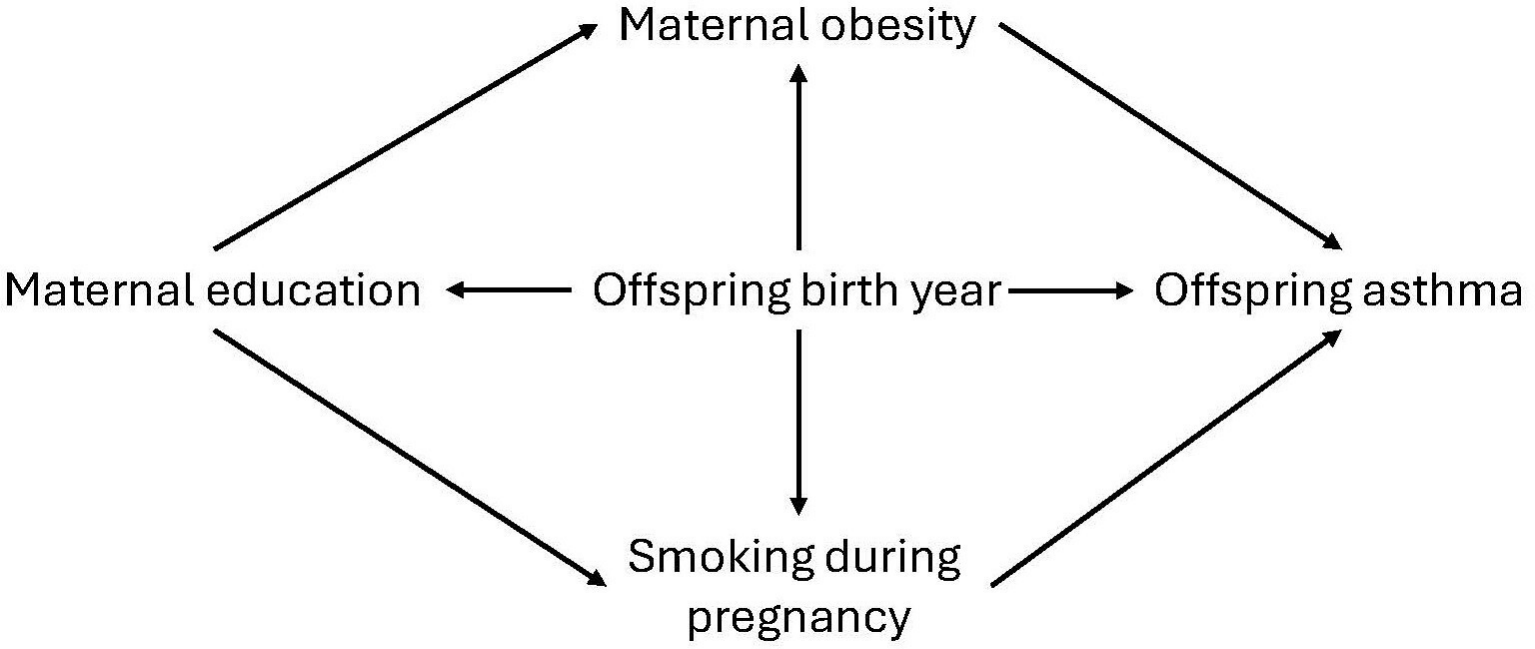
Directed acyclic graph illustrating the research question.

We selected covariates based on a directed acyclic graph (figure 1). We aimed to estimate the effect of education as a generic measure of SEP, rather than the effect of a certain number of years of education. Therefore, we did not include confounders to isolate the effect of education as such, for example, income and grandparental education. Consequently, we only included the year of the child’s birth as a covariate. Furthermore, we retrieved parity from the MBR, to be used for sensitivity analysis.

### Statistical methods

Bivariate exposure-mediator, exposure-outcome and mediator-outcome associations were estimated using logistic regression for obesity, SDP and asthma as dependent variables and linear regression with robust SEs for BMI as dependent variable.

For mediation analysis, there are two major approaches—the traditional/classical and the counterfactual/causal approach. The traditional approach does not allow for the estimation of indirect (mediated) effects when the mediator is dichotomous or when there is interaction between the exposure and the mediator, whereas the counterfactual approach does.^23^ We therefore used the counterfactual approach by VanderWeele^24^ to estimate the total effect and the decomposition of the total effect into the natural direct effect, the pure indirect effect and the mediated interaction, where the pure mediated effect and the mediated interaction are the components that can be attributed to the mediator. The pure mediated effect is the effect of the mediator on the outcome that is the same for both exposed and unexposed, while the mediated interaction is an effect of the mediator that is there only for the exposed.

To explore the mediated interaction further, we used logistic regression to estimate the interaction between the exposure and the mediator, with interaction terms parametrised to give separate estimates of the mediator-outcome ORs for the different levels of maternal education.

As sensitivity analyses, we repeated all analyses in the subgroups of first-born and non-first-born children, respectively. The mediation analyses were also performed stratified by sex.

Both unadjusted models and models adjusted for birth year as a categorical variable, with each year as a category, were presented. When BMI was used as an independent variable, it was modelled as a linear predictor. The only variables with missing data were maternal BMI/obesity and SDP. Complete case analysis was used rather than imputation due to low levels of missingness in combination with no difference in levels of missingness between the exposure groups. The mediation analyses were performed using the SAS procedure CAUSALMED, while Stata V.18.0 was used for bivariate associations and interaction analysis. The statistical analysis plan and analysis code can be retrieved from OSF (osf.io), DOI 10.17605/OSF.IO/KSVPQ.

## Results

In this cohort of 1 265 933 children, 49% had mothers with secondary school education or less. This proportion decreased with time. The prevalence of obesity was higher among mothers with secondary versus tertiary education (15.9% vs 8.1%, table 1) and likewise the mean BMI was higher among the mothers with secondary education compared with tertiary (25.5 vs 24.1, data not shown). Similarly, SDP was more common in the secondary education group compared with tertiary education (10.4% vs 1.2%, table 1).

**Table 1:** Description of the cohort of n=1 265 933 children and their mothers

	Mother’s education			
	Secondary education		Tertiary education	
	No.	%	No.	%
N	621 720		644 213	
Maternal obesity				
No	479 466	77.1	546 765	84.9
Yes	98 781	15.9	51 929	8.1
Missing	43 473	7.0	45 519	7.1
Smoking during pregnancy				
No	532 935	85.7	610 845	94.8
Yes	64 473	10.4	7719	1.2
Missing	24 312	3.9	25 649	4.0
Year of child’s birth				
2006	52 529	8.4	46 253	7.2
2007	52 376	8.4	48 079	7.5
2008	52 182	8.4	50 208	7.8
2009	51 644	8.3	51 847	8.0
2010	53 143	8.5	55 170	8.6
2011	51 278	8.2	53 573	8.3
2012	51 232	8.2	54 423	8.4
2013	50 732	8.2	55 468	8.6
2014	52 084	8.4	56 374	8.8
2015	51 967	8.4	56 919	8.8
2016	51 591	8.3	57 724	9.0
2017	50 962	8.2	58 175	9.0
Sex				
Male	319 234	51.3	331 661	51.5
Female	302 486	48.7	312 552	48.5
First-born				
No	366 255	58.9	351 881	54.6
Yes	255 465	41.1	292 332	45.4
Offspring asthma during the third year of life				
No	568 292	91.4	595 257	92.4
Yes	53 428	8.6	48 956	7.6
Offspring asthma during the sixth year of life				
No	567 303	91.2	589 718	91.5
Yes	49 200	7.9	47 510	7.4
Excluded*	5217	0.8	6985	1.1
Emigrated before age 6 years				
No	616 682	99.2	637 355	98.9
Yes	5038	0.8	6858	1.1
Dead before age 6 years				
No	621 541	100.0	644 086	100.0
Yes	179	0.0	127	0.0

* Excluded from analyses on asthma during the sixth year of life due to emigration or death before age 6 years.

Estimated bivariate associations between the exposure variable maternal education; the mediators maternal obesity, BMI and SDP and the outcome variables asthma during the third and sixth years of life in the child are displayed in table 2. There were strong associations between maternal education and both mediators, for example, unadjusted OR 2.17 (95% CI 2.14 to 2.19) for obesity and OR 9.57 (95% CI 9.35 to 9.81) for SDP. The unadjusted association between the exposure variable maternal education and the outcome during the third year of life (OR 1.14, 95% CI 1.13 to 1.16) was stronger than the association with the outcome during the sixth year (OR 1.08, 95% CI 1.06 to 1.09). Likewise, the unadjusted ORs with SDP were stronger with asthma during the third year of life (OR 1.36, 95% CI 1.32 to 1.39) than the sixth year (OR 1.13, 95% CI 1.09 to 1.16), while they were very similar for obesity (asthma during the third year: OR 1.33 95% CI 1.30 to 1.35; asthma during the sixth year: OR 1.34, 95% CI 1.32 to 1.37) and BMI (asthma during the third year: OR 1.13, 95% CI 1.12 to 1.14; asthma during the sixth year: OR 1.13, 95% CI 1.13 to 1.14). Adjustment for birth year rendered only marginal changes in estimates. Estimates for the subgroups of first-born and non-first-born were similar, although the association between maternal education and obesity/BMI was slightly weaker in the first-born group (online supplemental table s1).

**Table 2:** ORs and linear regression coefficients (β) with 95% CIs for bivariate associations between exposure, mediators and outcomes

Independent variable	Dependent variable	Unadjusted	Adjusted*
		OR or β (95% CI)	OR or β (95% CI)
Exposure-mediator associations			
Low maternal education	Obesity†	2.17 (2.14 to 2.19)	2.19 (2.16 to 2.21)
Low maternal education	BMI‡	1.39 (1.37 to 1.40)	1.40 (1.38 to 1.42)
Low maternal education	Smoking during pregnancy†	9.57 (9.35 to 9.81)	9.48 (9.26 to 9.71)
Exposure-outcome associations			
Low maternal education	Asthma during third year of life†	1.14 (1.13 to 1.16)	1.15 (1.13 to 1.16)
Low maternal education	Asthma during sixth year of life†	1.08 (1.06 to 1.09)	1.09 (1.07 to 1.10)
Mediator-outcome associations			
Obesity	Asthma during third year of life†	1.33 (1.30 to 1.35)	1.32 (1.30 to 1.34)
Obesity	Asthma during sixth year of life†	1.34 (1.32 to 1.37)	1.33 (1.31 to 1.36)
BMI§	Asthma during third year of life†	1.13 (1.12 to 1.14)	1.13 (1.12 to 1.14)
BMI§	Asthma during sixth year of life†	1.13 (1.13 to 1.14)	1.13 (1.12 to 1.14)
Smoking during pregnancy	Asthma during third year of life†	1.36 (1.32 to 1.39)	1.38 (1.34 to 1.41)
Smoking during pregnancy	Asthma during sixth year of life†	1.13 (1.09 to 1.16)	1.15 (1.12 to 1.18)

* Adjusted for birth year.

† Logistic regression, OR shown.

‡ Linear regression, regression coefficient shown.

§ Per five units higher BMI.

BMI, body mass index.

**Figure 2:**
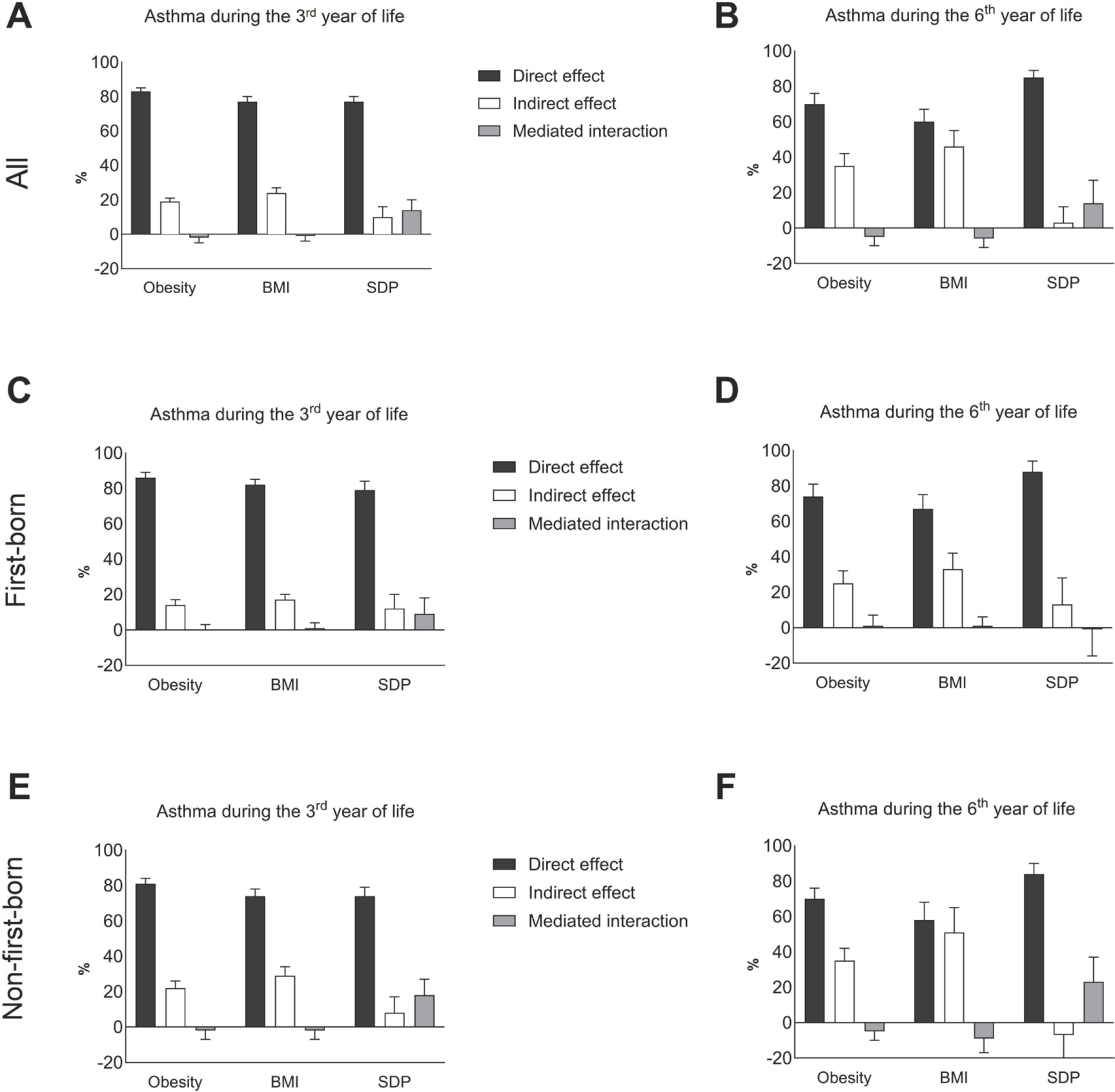
Results of mediation analysis showing percentage of associations between the exposure (maternal education) and the outcome (offspring asthma) during the third (**A, C and E**) and the sixth (**B, D and F**) years of life explained by the direct effect, indirect effect of each of the mediators (obesity, BMI and SDP) and the interaction between the exposure and the mediators. Results are shown for all children (**A–B**), first-born (**C–D**) and non-first-born (**E–F**). BMI, body mass index; SDP, smoking during pregnancy.

Results from mediation analyses are displayed in figure 2 and online supplemental table s2. Out of the excess risk of asthma during the third year of life among children whose mothers had low education, 19% could be explained by obesity, 24% by BMI and 10% by SDP. The corresponding figures for asthma during the sixth year of life were 35% (obesity), 46% (BMI) and 3% (SDP). The results also indicated that the association was partly explained by an interaction between the exposure, maternal education and the mediators, particularly for SDP. The interaction between maternal education and SDP explained 14% of the association both during the third and the sixth year of life, while the interaction with obesity/BMI was small and in the opposite direction (−2% with BMI and −1% with obesity for asthma during the third year of life and −5% with BMI and −6% with obesity for asthma during the sixth year of life). Results stratified by first-born and non-first-born indicated that a slightly larger proportion of the association was explained by mediation by obesity, BMI and SDP or interaction between those mediators and the exposure in non-first-born than in first-born children. For obesity and BMI, a larger proportion of the association was explained by mediation, whereas for SDP, a larger proportion was explained by interaction between maternal education and SDP (figure 2). Mediation analyses stratified by sex are presented in online supplemental table s3.

Table 3 displays the interaction between the exposure (maternal education) and the mediators (obesity, BMI and SDP), that is, how the associations between the mediators and the outcome differ by maternal education. The associations between obesity or BMI and asthma were slightly stronger for children with highly educated mothers (eg, asthma during the third year of life and obesity OR 1.35, 95% CI 1.30 to 1.39 for high education vs OR 1.28, 95% CI 1.21 to 1.35 for low education). The opposite was found for SDP, with a weaker association for highly educated mothers (eg, asthma during the third year of life OR 1.15, 95% CI 1.06 to 1.24 for high education vs OR 1.32, 95% CI 1.28 to 1.36 for low education). When stratifying by first-born and non-first-born, the results mirror the pattern from the mediation analyses in that the exposure-mediator interaction was more pronounced for non-first-born than for first-born, particularly for SDP (online supplemental table s2).

**Table 3:** Interactions between maternal education and the mediators: maternal obesity, BMI and SDP

	Maternal education		P value for interaction
	Low	High	
All			
Asthma during the third year of life			
Obesity	1.27 (1.24 to 1.30)	1.34 (1.29 to 1.38)	0.010
BMI*	1.11 (1.10 to 1.12)	1.13 (1.12 to 1.14)	0.014
SDP	1.33 (1.30 to 1.37)	1.16 (1.07 to 1.25)	0.0008
Asthma during the sixth year of life			
Obesity	1.29 (1.26 to 1.32)	1.36 (1.32 to 1.41)	0.006
BMI*	1.12 (1.11 to 1.13)	1.14 (1.13 to 1.15)	0.002
SDP	1.13 (1.09 to 1.16)	1.02 (0.94 to 1.11)	0.036
First-born			
Asthma during the third year of life			
Obesity	1.31 (1.26 to 1.36)	1.36 (1.30 to 1.43)	0.21
BMI*	1.13 (1.12 to 1.15)	1.14 (1.12 to 1.16)	0.50
SDP	1.31 (1.26 to 1.37)	1.20 (1.07 to 1.35)	0.17
Asthma during the sixth year of life			
Obesity	1.37 (1.32 to 1.42)	1.37 (1.31 to 1.44)	0.88
BMI*	1.15 (1.13 to 1.16)	1.15 (1.14 to 1.17)	0.65
SDP	1.11 (1.06 to 1.16)	1.12 (1.00 to 1.27)	0.83
Non-first-born			
Asthma during the third year of life			
Obesity	1.25 (1.22 to 1.29)	1.32 (1.26 to 1.37)	0.044
BMI*	1.10 (1.09 to 1.11)	1.12 (1.11 to 1.14)	0.034
SDP	1.35 (1.31 to 1.40)	1.12 (1.01 to 1.25)	0.001
Asthma during the sixth year of life			
Obesity	1.27 (1.23 to 1.31)	1.37 (1.32 to 1.43)	0.003
BMI*	1.11 (1.10 to 1.12)	1.14 (1.12 to 1.16)	0.004
SDP	1.14 (1.10 to 1.18)	0.93 (0.83 to 1.06)	0.002

All analyses are adjusted for birth year.

* Per five units higher BMI.

BMI, body mass index; SDP, smoking during pregnancy.

## Discussion

In this population-based cohort study of 1.3 million children, we found that 20%–30% of the excess risk of offspring asthma during the third and sixth years of life among children of mothers with low education was explained by the mediator maternal obesity or BMI and 15%–20% was explained by SDP. This was partly due to interactions between maternal education and the mediators, indicating that obesity and BMI had stronger association with asthma in the offspring if the mother had high education, while the association between SDP and offspring asthma was stronger if the mother had low education. Our results also indicated that for first-born children, there was a slightly larger proportion of the excess risk in the low education group that could be explained by obesity or BMI and SDP as compared with non-first-born children.

Our findings that maternal obesity or BMI and SDP mediate some of the association between maternal SEP and offspring asthma during the third and sixth years of life are supported by findings in two cohort studies with children aged 7–14 years, one showing significant mediation by SDP^25^ and the other 37% mediation by a block of perinatal factors including, among others, maternal SDP and prepregnancy BMI.^26^ A study with wheeze as outcome found that the association between SEP and wheeze was partly explained by SDP,^27^ while another study with breathlessness and wheeze at age 4 years as outcomes found that a block of prenatal factors partly explained the associations between SEP and breathlessness, but not wheeze.^28^ To our knowledge, we are the first to quantify the role of the modifiable risk factors maternal obesity, BMI or SDP in the association between SEP and asthma. Our results indicate that supporting women of reproductive age with lower SEP to maintain or achieve a healthy weight and to abstain from tobacco smoking could reduce their own morbidity and health inequities in childhood for their offspring.

Our subgroup analyses indicated that maternal obesity or BMI and smoking may be more important in subsequent pregnancies than in the first. Thus, the time after a first pregnancy could be an opportunity to support women with unhealthy weight and tobacco dependency. Future research should focus on intervention programmes targeting women during and between pregnancies.

A major strength of this study is the unique longitudinal registry data covering the entire population, which enabled us to link parental socioeconomic information with information on exposures in pregnancy and offspring respiratory health, ensuring generalisability to the general population. Since the data are registered prospectively by authorities and health professionals, recall bias is avoided. However, using data not collected for research purposes also means that we could not control the way data were collected, for example, the exact timepoint in pregnancy when the women’s BMI was measured and the way questions about smoking are asked. Other limitations of this study include the measurement error in the asthma variables. As a consequence of not having information about primary care visits, but only hospital-based specialist care and drug prescription dispenses, we may misclassify mild asthma treated in primary care without or with very few dispensed prescriptions as no asthma. Furthermore, the use of education as a proxy of SEP could be discussed but is generally considered a robust measure of SEP, which is widely used. When focusing on SEP in a broader sense, it also has the advantage of being influenced by the family background and SEP in the previous generation and can thereby be viewed as a generic measure of SEP. Finally, mediation analysis assumes no unmeasured confounding between exposure and mediator, exposure and outcome and between mediator and outcome. We cannot exclude unmeasured confounding from health behaviour, which may increase the risk of obesity and smoking, while its effect on offspring asthma is unclear.

In conclusion, our results showed 15% and 9% increased odds of asthma in children of mothers with low education compared with high education during the third and sixth years of life, respectively. Around 20%–30% of the excess risk of offspring asthma among mothers with low education compared with high education could be explained by obesity or BMI through mediation or mediator-exposure interaction, while 15%–20% could be explained by SDP. Our results highlight the role of intervention programmes supporting young women and mothers-to-be to healthy weight and to abstain from tobacco smoking, in particular during pregnancy, in order to reduce child morbidity and improve health equity in childhood.

## Supplementary material

10.1136/thorax-2025-223330 online supplemental file 1

10.1136/thorax-2025-223330 online supplemental file 2

## Data Availability

Data are available on reasonable request. Data may be obtained from a third party and are not publicly available.
