# Supplementary material for: Maternal BMI and smoking partly explain the association between maternal socio-economic position and offspring asthma
Source: Thorax. 2025 Oct 28;81(5):e223330. doi: 10.1136/thorax-2025-223330 (PMC13151482; doi:10.1136/thorax-2025-223330)
Supplement: online supplemental file 1 [file thorax-81-5-s001.pdf]

Table S1. Odds ratios (OR) and linear regression coefficients (β) with 95% confidence intervals (CI) for bivariate associations between exposure, mediators and outcomes

Independent variable	Dependent variable	First born Unadjusted OR or β (95% CI)	First born Adjusted[c] OR or β (95% CI)	Non-first born Unadjusted OR or β (95% CI)	Non-first born Adjusted[c] OR or β (95% CI)
**Exposure-mediator associations**					
Low maternal education	Obesity[a]	1.95 [1.91,1.99]	1.97 [1.93,2.00]	2.27 [2.24,2.30]	2.29 [2.26,2.32]
Low maternal education	BMI[b]	0.99 [0.97,1.02]	1.01 [0.98,1.03]	1.62 [1.60,1.65]	1.64 [1.61,1.66]
Low maternal education	Smoking during pregnancy[a]	9.65 [9.31,10.00]	9.55 [9.22,9.90]	9.52 [9.22,9.83]	9.43 [9.14,9.74]
**Exposure-outcome associations**					
Low maternal education	Asthma during the 3rd year of life[a]	1.16 [1.13,1.18]	1.16 [1.14,1.19]	1.13 [1.11,1.15]	1.14 [1.12,1.16]
Low maternal education	Asthma during the 6th year of life[a]	1.08 [1.06,1.10]	1.09 [1.07,1.11]	1.08 [1.06,1.10]	1.09 [1.07,1.11]
**Mediator- outcome associations**					
Obesity	Asthma during the 3rd year of life[a]	1.37 [1.33,1.41]	1.36 [1.32,1.40]	1.31 [1.28,1.34]	1.30 [1.27,1.33]
Obesity	Asthma during the 6th year of life[a]	1.40 [1.36,1.44]	1.39 [1.34,1.43]	1.33 [1.30,1.36]	1.32 [1.29,1.35]
BMI[d]	Asthma during the 3rd year of life[a]	1.15 [1.14,1.16]	1.14 [1.13,1.16]	1.12 [1.11,1.13]	1.12 [1.11,1.13]
BMI[d]	Asthma during the 6th year of life[a]	1.16 [1.15,1.17]	1.16 [1.14,1.17]	1.13 [1.12,1.14]	1.12 [1.11,1.13]
Smoking during pregnancy	Asthma during the 3rd year of life[a]	1.35 [1.30,1.40]	1.37 [1.32,1.42]	1.37 [1.32,1.41]	1.38 [1.34,1.43]
Smoking during pregnancy	Asthma during the 6th year of life[a]	1.12 [1.08,1.17]	1.15 [1.10,1.19]	1.13 [1.09,1.17]	1.15 [1.11,1.19]

[a] Logistic regression, odds ratio (OR) shown

[b] Linear regression, regression coefficient (β) shown

[c] Adjusted for birth year

[d] OR per 5 units higher BMI

Table s2. Results from mediation analysis of the association between maternal education and offspring asthma with obesity, BMI and smoking during pregnancy (SDP) as mediators, adjusted for birthyear for all children and stratified by first born and non-first born in a cohort of N=1,265,933 children.

	Odds ratio (95% CI)			Excess relative risk in % (95% CI)				Percent (95% CI)		
	**TE**	**NDE**	**NIE**	**TE**	**NDE**	**PIE**	**Mediated inter-action**	**Direct effect**	**In-direct effect**	**Mediated inter-action**
**All**										
**Asthma during the 3rd year of life**										
Obesity	1.15	1.12	1.02	14.9	12.4	2.8	-0.3	83	19	-2
	(1.13, 1.16)	(1.11, 1.14)	(1.02, 1.02)	(13.4, 16.5)	(11.9, 14.0)	(2.4, 3.1)	(-0.7, 0.2)	(81,85)	(16,21)	(-5, 1)
BMI	1.15	1.11	1.03	14.7	11.3	3.5	-0.2	77	24	-1
	(1.13, 1.16)	(1.10, 1.13)	(1.03, 1.03)	(13.1, 16.2)	(9.8, 12.8)	(3.2, 3.8)	(-0.6, 0.3)	(74,80)	(21,27)	(-4, 1)
SDP	1.15	1.11	1.03	14.9	11.4	1.5	2.0	77	10	14
	(1.13, 1.16)	(1.10, 1.13)	(1.03, 1.03)	(13.3, 16.4)	(9.9, 12.9)	(0.6, 2.4)	(1.1, 3.0)	(73,80)	(4, 16)	(7, 20)
**Asthma during the 6th year of life**										
Obesity	1.09	1.06	1.02	8.5	6.0	3.0	-0.4	70	35	-5
	(1.07, 1.10)	(1.05, 1.07)	(1.02, 1.03)	(7.1, 10.0)	(4.5, 7.5)	(2.7, 3.3)	(-0.9, -0.0)	(64,76)	(28,42)	(-10, -0)
BMI	1.08	1.05	1.03	8.2	4.9	3.8	-0.5	60	46	-6
	(1.07, 1.10)	(1.03, 1.06)	(1.03, 1.03)	(6.8, 9.7)	(3.5, 6.4)	(3.4, 4.1)	(-0.9, -0.0)	(53,67)	(37,55)	(-11, -1)
SDP	1.08	1.07	1.01	8.4	7.1	0.2	1.0	85	3	14
	(1.07, 1.10)	(1.06, 1.09)	(1.01, 1.02)	(6.9, 9.8)	(5.6, 8.6)	(-0.6, 1.0)	(0.1, 2.0)	(80,89)	(-7, 12)	(2, 27)
**First born**										
**Asthma during the 3rd year of life**										
Obesity	1.16	1.14	1.03	16.2	14.1	2.2	-0.1	86	14	-0
	(1.14, 1.19)	(1.12, 1.15)	(1.02, 1.03)	(14.9, 18.6)	(11.7, 16.4)	(1.8, 2.6)	(-0.6, 0.5)	(84,89)	(11,17)	(-4, 3)
BMI	1.16	1.13	1.03	16.0	13.1	2.7	0.2	82	17	1
	(1.14, 1.18)	(1.08, 1.15)	(1.03, 1.03)	(13.6, 18.3)	(10.8, 15.4)	(2.4, 3.1)	(-0.3, 0.6)	(79,85)	(14,20)	(-2, 4)
SDP	1.16	1.13	1.03	16.2	12.9	1.9	1.4	79	12	9
	(1.14, 1.19)	(1.11, 1.15)	(1.02, 1.03)	(13.9, 18.5)	(10.6, 15.2)	(0.6, 3.3)	(-0.0, 2.9)	(75,84)	(3, 20)	(-0, 18)

**Asthma during the 6th year of life**										
Obesity	1.09	1.07	1.02	9.3	6.9	2.3	0.1	74	25	1
	(1.07, 1.12)	(1.05, 1.09)	(1.02, 1.03)	(7.1, 11.6)	(4.7, 9.1)	(1.9, 2.7)	(-0.4, -0.6)	(67,81)	(17,32)	(-4, 7)
BMI	1.09	1.06	1.03	9.0	6.0	2.9	0.1	67	33	1
	(1.07, 1.11)	(1.04, 1.08)	(1.03, 1.03)	(6.8, 11.2)	(3.8, 8.2)	(2.6, 3.3)	(-0.3, 0.5)	(58,75)	(24,42)	(-4, 6)
SDP	1.09	1.08	1.01	8.9	7.8	1.1	-0.1	88	13	-1
	(1.07, 1.11)	(1.06, 1.10)	(1.01, 1.02)	(6.7, 11.1)	(5.6, 10.0)	(-0.1, 2.5)	(-1.4, 1.3)	(81,94)	(-2, 28)	(-16, 15)
**Non-first born**										
**Asthma during the 3rd year of life**										
Obesity	1.14	1.11	1.02	13.9	11.2	3.0	-0.3	81	22	-2
	(1.12, 1.16)	(1.10, 1.14)	(1.02, 1.03)	(11.9, 16.0)	(9.2, 13.2)	(2.5, 3.5)	(-0.9, 0.3)	(77,84)	(17,26)	(-7, 2)
BMI	1.14	1.10	1.03	13.7	10.1	3.9	-0.3	74	29	-2
	(1.12, 1.16)	(1.08, 1.12)	(1.03, 1.04)	(11.7, 15.7)	(8.1, 12.1)	(3.4, 4.4)	(-0.9, 0.3)	(69,78)	(23,34)	(-7, 2)
SDP	1.14	1.10	1.03	13.8	10.2	1.1	2.5	74	8	18
	(1.12, 1.16)	(1.08, 1.12)	(1.03, 1.04)	(11.8, 15.8)	(8.2, 12.2)	(-0.0, 2.3)	(1.2, 3.7)	(69,79)	(-0, 17)	(9, 27)
**Asthma during the 6th year of life**										
Obesity	1.09	1.06	1.02	8.5	6.0	3.0	-0.4	70	35	-5
	(1.07, 1.10)	(1.05, 1.07)	(1.02, 1.03)	(7.1, 10.0)	(4.5, 7.5)	(2.7, 3.3)	(-0.9, -0.0)	(64,76)	(28,42)	(-10, -0)
BMI	1.08	1.05	1.03	8.5	4.9	4.3	-0.8	58	51	-9
	(1.06, 1.10)	(1.03, 1.07)	(1.03, 1.04)	(6.5, 10.5	(2.9, 6.9)	(3.8, 4.8)	(-1.4, -0.1)	(48,68)	(37, 65)	(-17, -1)
SDP	1.09	1.07	1.01	8.8	7.4	-0.6	2.0	84	-7	23
	(1.07, 1.11)	(1.05, 1.09)	(1.01, 1.02)	(6.8, 10.7)	(5.4, 9.4)	(-1.7, 0.5)	(0.9, 3.2)	(78, 90)	(-20, 5)	(9, 37)

All analyses are adjusted for birthyear.

TE = Total effect, NDE = Natural direct effect, NIE = Natural indirect effect, PIE = Pure indirect effect

Table s3. Sex-specific results from mediation analysis of the association between maternal education and offspring asthma with obesity, BMI and smoking during pregnancy (SDP) as mediators, adjusted for birthyear in a cohort of N=650,895 boys and N=615,038 girls.

	Odds ratio (95% CI)			Excess relative risk in % (95% CI)				Percent (95% CI)		
	TE	NDE	NIE	TE	NDE	PIE	Mediated inter-action	Direct effect	In-direct effect	Mediated inter-action
**Boys**										
**Asthma during the 3rd year of life**										
Obesity	1.14	1.11	1.02	13.7	11.2	2.8	-0.4	82	21	-3
	(1.12, 1.16)	(1.09, 1.13)	(1.02, 1.02)	(11.7, 15.6)	(9.3, 13.1)	(2.4, 3.3)	(-0.9, 0.1)	(79,85)	(16,25)	(-7, 1)
BMI	1.13	1.10	1.03	13.4	10.2	3.5	-0.3	76	26	-2
	(1.11, 1.15)	(1.08, 1.12)	(1.03, 1.03)	(11.5, 15.4)	(8.3, 12.1)	(3.1, 3.9)	(-0.8, 0.2)	(72,80)	(21,31)	(-6, 2)
SDP	1.14	1.10	1.03	13.5	10.3	1.5	1.8	76	11	13
	(1.12, 1.15)	(1.08, 1.12)	(1.03, 1.03)	(11.6, 15.4)	(8.4, 12.2)	(0.4, 2.6)	(0.5, 3.0)	(72,81)	(2, 19)	(4, 22)
**Asthma during the 6th year of life**										
Obesity	1.07	1.05	1.02	7.0	4.6	3.1	-0.7	66	44	-10
	(1.05, 1.09)	(1.03, 1.07)	(1.02, 1.03)	(5.1, 8.9)	(2.8, 6.5)	(2.7, 3.5)	(-1.2, -0.1)	(56,76)	(31,58)	(-18, -2)
BMI	1.07	1.04	1.03	6.7	3.7	3.6	-0.5	55	54	-9
	(1.05, 1.09)	(1.02, 1.06)	(1.03, 1.03)	(4.9, 8.7)	(1.9, 5.6)	(3.2, 4.1)	(-1.1, -0.1)	(42,68)	(38,70)	(-17, -0)
SDP	1.07	1.06	1.01	7.0	5.8	0.3	0.9	82	5	13
	(1.05, 1.09)	(1.04, 1.08)	(1.01, 1.02)	(5.1, 8.8)	(3.9, 7.6)	(-0.7, 1.4)	(-0.3, 2.1)	(75,90)	(-11, 20)	(-4, 30)
**Girls**										
**Asthma during the 3rd year of life**										
Obesity	1.17	1.15	1.02	17.3	14.6	2.7	-0.0	85	16	-0
	(1.15, 1.20)	(1.12, 1.17)	(1.02, 1.03)	(14.8, 19.8)	(12.1,17.1)	(2.1, 3.2)	(-0.7, 0.7)	(81,88)	(12,19)	(-4, 4)
BMI	1.17	1.13	1.03	17.0	13.4	3.5	0.0	79	21	0
	(1.14, 1.19)	(1.11, 1.16)	(1.03, 1.04)	(14.5, 19.5)	(11.0,15.9)	(3.0, 4.0)	(-0.6, 0.7)	(75,83)	(17,25)	(-4, 4)
SDP	1.17	1.13	1.03	17.3	13.4	1.5	2.4	78	9	14
	(1.15, 1.20)	(1.11, 1.16)	(1.03, 1.04)	(14.9, 19.8)	(11.0,15.9)	(0.1, 2.9)	(0.9, 3.9)	(73,82)	(1, 17)	(5, 23)
**Asthma during the 6th year of life**										

Obesity	1.11	1.08	1.03	11.1	8.3	2.8	-0.0	75	25	-0
	(1.09, 1.14)	(1.06, 1.11)	(1.02, 1.03)	(8.7, 13.5)	(5.9, 10.7)	(2.3, 3.4)	(-0.7, 0.7)	(69,81)	(18,33)	(-6, 6)
BMI	1.11	1.07	1.04	10.8	7.1	3.9	-0.3	66	37	-3
	(1.08, 1.13)	(1.05, 1.09)	(1.03, 1.04)	(8.4, 13.2)	(4.8, 9.5)	(3.4, 4.4)	(-1.0, 0.3)	(58,74)	(27, 46)	(-9, 3)
SDP	1.11	1.09	1.01	10.7	9.4	0.0	1.3	88	0	12
	(1.08, 1.13)	(1.07, 1.12)	(1.01, 1.02)	(8.4, 13.1)	(7.0, 11.8)	(-1.3, 1.3)	(-0.1, 2.7)	(82, 93)	(-12, 12)	(-1, 25)

All analyses are adjusted for birthyear.

TE = Total effect, NDE = Natural direct effect, NIE = Natural indirect effect, PIE = Pure indirect effect
