# Supplementary material for: Maternal BMI and smoking partly explain the association between maternal socio-economic position and offspring asthma
Source: Thorax. 2025 Oct 28;81(5):e223330. doi: 10.1136/thorax-2025-223330 (PMC13151482; doi:10.1136/thorax-2025-223330)
Supplement: online supplemental file 2 [file thorax-81-5-s002.pptx]

## Slide 1
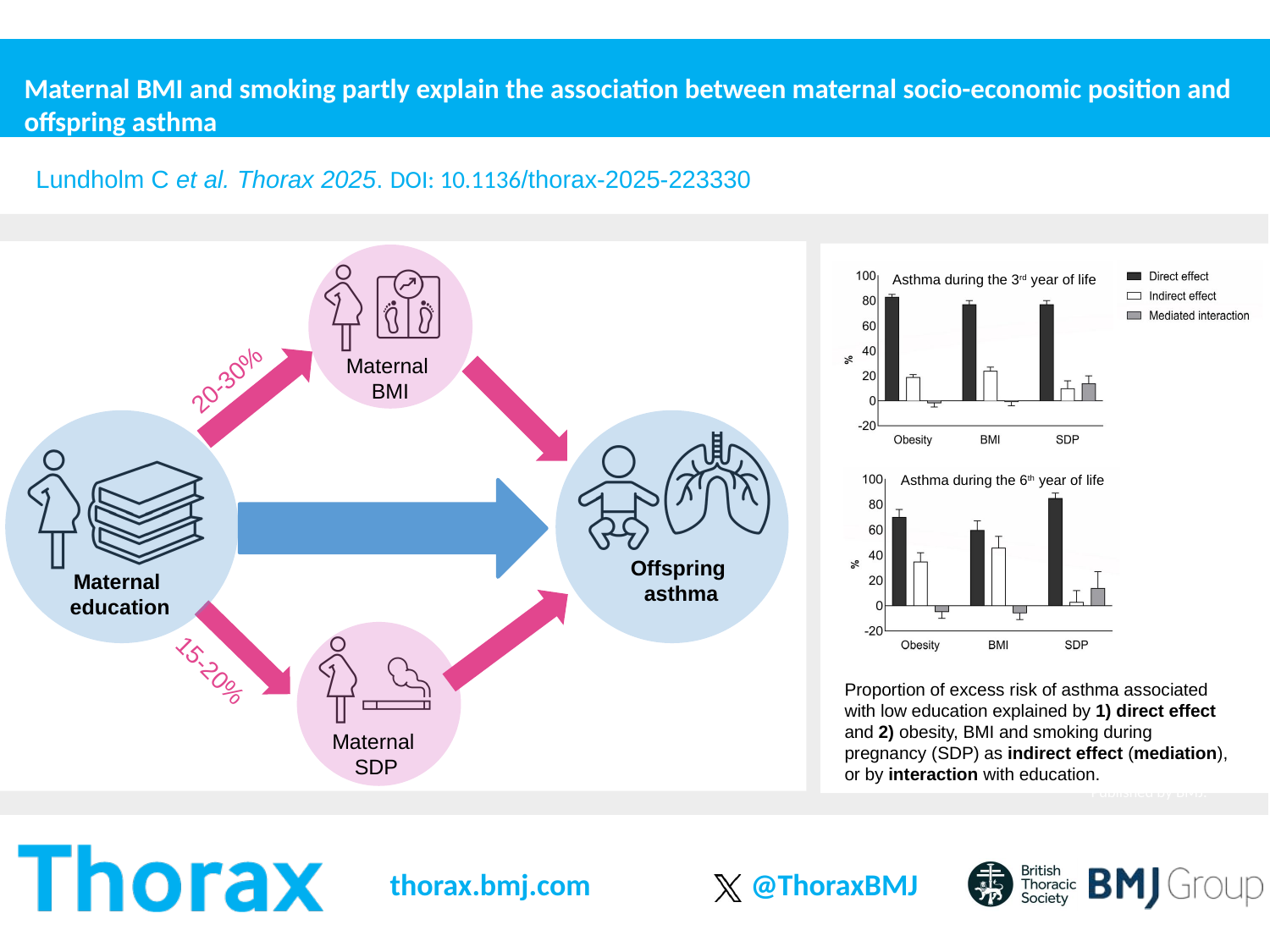

Maternal BMI and smoking partly explain the association between maternal socio-economic position and offspring asthma
Lundholm C et al. Thorax 2025. DOI: 10.1136/thorax-2025-223330
Asthma during the 3rd year of life
Manuscript Title
Maternal
BMI
20-30%
Asthma during the 6th year of life
Offspring
asthma
Maternal
education
15-20%
Proportion of excess risk of asthma associated with low education explained by 1) direct effect and 2) obesity, BMI and smoking during pregnancy (SDP) as indirect effect (mediation), or by interaction with education.
© Author(s) (or their employer(s) 2019. Re-use permitted under CC BY. Published by BMJ.
Maternal
SDP
thorax.bmj.com @ThoraxBMJ
